# Cell-based high-throughput screening of polysaccharide biosynthesis hosts

**DOI:** 10.1186/s12934-021-01555-w

**Published:** 2021-03-05

**Authors:** Zi-Xu Liu, Si-Ling Huang, Jin Hou, Xue-Ping Guo, Feng-Shan Wang, Ju-Zheng Sheng

**Affiliations:** 1 Key Laboratory of Chemical Biology of Natural Products (Ministry of Education), Institute of Biochemical and Biotechnological Drug, School of Pharmaceutical Sciences, Cheeloo College of Medicine, Shandong University, Jinan, 250012 China; 2 Bloomage BioTechnology Corp., Ltd., Jinan, 250010 China; 3 The State Key Laboratory of Microbial Technology, Shandong University, Jinan, 250100 China; 4 National Glycoengineering Research Center, Shandong University, Jinan, 250012 China

**Keywords:** Polysaccharide, Glycosyltransferase, High-throughput screening, Fluorescently-labeled substrate, Biosensor

## Abstract

Valuable polysaccharides are usually produced using wild-type or metabolically-engineered host microbial strains through fermentation. These hosts act as cell factories that convert carbohydrates, such as monosaccharides or starch, into bioactive polysaccharides. It is desirable to develop effective in vivo high-throughput approaches to screen cells that display high-level synthesis of the desired polysaccharides. Uses of single or dual fluorophore labeling, fluorescence quenching, or biosensors are effective strategies for cell sorting of a library that can be applied during the domestication of industrial engineered strains and metabolic pathway optimization of polysaccharide synthesis in engineered cells. Meanwhile, high-throughput screening strategies using each individual whole cell as a sorting section are playing growing roles in the discovery and directed evolution of enzymes involved in polysaccharide biosynthesis, such as glycosyltransferases. These enzymes and their mutants are in high demand as tool catalysts for synthesis of saccharides in vitro and in vivo. This review provides an introduction to the methodologies of using cell-based high-throughput screening for desired polysaccharide-biosynthesizing cells, followed by a brief discussion of potential applications of these approaches in glycoengineering.

## Background

Bacterial polysaccharides are cell-protective macromolecular polymers, polymerized from various monosaccharides through specific glyosidic bonds [1–6]. Because of their unique physical, chemical and rheological properties and good biosafety, natural bacterial polysaccharides have extensive applications in many fields, including as drugs, pharmaceutical materials, bioengineering materials, food additives, and microbial flocculants [7–16]. In addition, natural capsular polysaccharides of some bacteria that are consistent with the backbone structure of mammalian polysaccharides have the potential to be used as synthetic precursors to realize large-scale preparation of active human polysaccharides, whose efficient and safe preparation has become a research hotspot. Glycosaminoglycan (GAG) drugs have become a typical representative of this research direction [17–24]. For example, bacterial polysaccharides consistent with the unsulfated modified polysaccharide skeletons of chondroitin sulfate and heparin were efficiently prepared by fermentation-based preparation of *Escherichia coli*-derived K4 and K5, following which the polysaccharides were sulfated in vitro to form non-animal GAGs [25, 26]. However, the molecular weight of polysaccharides is high, their structures are complex, and the synthesis of these biomacromolecules in cells involves multiple enzymatic reactions. Moreover, the study of bacterial polysaccharide production in engineered microbial strains faces a common problem—the balance between cell growth and product synthesis [27, 28]. In addition, although many studies have been carried out on the synthesis pathways and carbohydrate chain structures of Gram-positive and Gram-negative bacterial polysaccharides, understanding of the mechanisms of regulation of bacterial carbohydrate chain synthesis still lags behind that of proteins and nucleic acids [29]. Therefore, as a tool for the domestication of polysaccharide-producing strains or to support genetic engineering, high-throughput screening methods for whole cells that have the ability to synthesize high levels of polysaccharides have significant research value.

Bacterial cells can also be used as effective carriers to realize the directed evolution of enzymes related to bacterial polysaccharide synthesis. With the rapid development of glycobiology and glycoengineering, carbohydrates have become an important source of leading compounds for drug discovery, and research and development of carbohydrate drugs shows increasing application potential and value [30, 31]. In vitro enzymatic synthesis of structurally defined oligosaccharides and glycoconjugates has leapt forward in recent years [32, 33]. Whether artificial carbohydrate-based drugs are chemo-enzymatically synthesized in vitro, or produced by engineered cells in vivo, efficient biocatalysts are in high demand. The cost of recombinant expression of bacterial proteins is usually lower than of animal proteins, making bacterial enzymes more suitable for synthetic approaches in vitro [34, 35]. Recently, a series of microbial enzymes, especially glycosyltransferases (GTs), that catalyze synthesis of saccharides with the same structures as human saccharides have been identified and their catalytic mechanism is being studied in depth [36–38]. Using protein engineering to modify wild-type GTs to obtain mutants with higher catalytic efficiency or novel substrate specificity is an effective means to improve productivity and expand the available structures of polysaccharide products [39–44]. An efficient directed-evolution platform for GTs would be of great significance for obtaining excellent enzymes with reduced effort [45]. High-throughput screening methods play a pivotal role in these platforms.

This review focuses on high-throughput screening methods based on single cells or single clones with different genotypes. Similar to the protein directed-evolution strategy that simulates Darwinian evolution in a test tube, the cell-based high-throughput screening of polysaccharide biosynthesis hosts is generally divided into two steps: (i) a large number of mutations are engineered, either as random mutations in the entire genetic material of a cell, or as changes in the sequence of a gene that encodes a particular enzyme; (ii) cells with changed levels of polysaccharide synthesis are sorted to realize the evolution at the cellular level. The differences in polysaccharide synthesis ability of individual cells can be identified quickly by means of fluorescence signals, growth differences, and other methods that do not require a lot of labor. This review focuses on part ii of the process.

## Screening based on fluorescently-labeled substrates

**Fig. 1:**
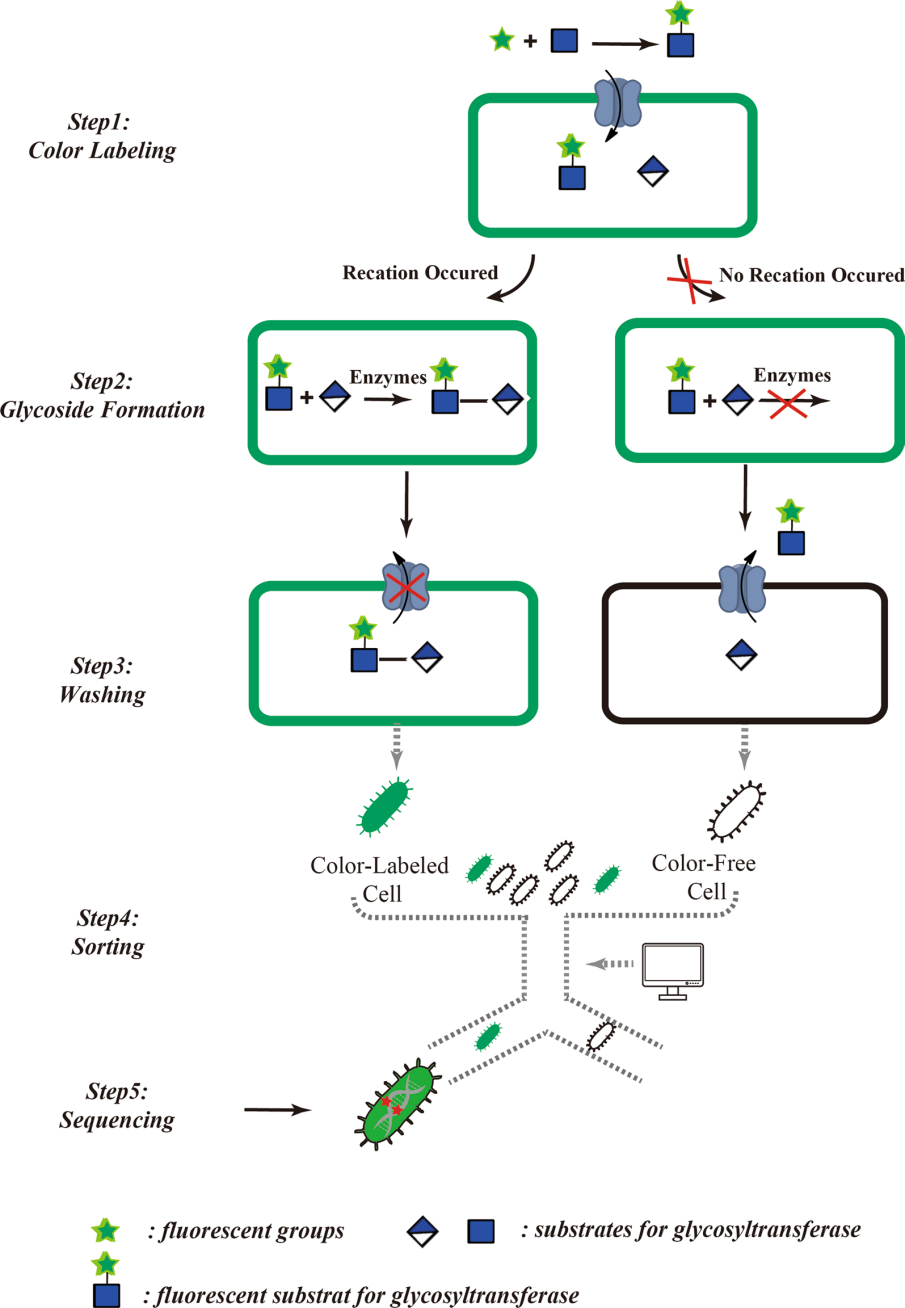
Schematic view of cell-based high-throughput screening strategies based on fluorescently-labeled substrates. In the original strategy, a single fluorescent substrate was used to screen a library of mutant sialyltransferases. First, fluorescently-labeled monosaccharides are transported into the cell by a transmembrane sugar transport protein (step 1). After an incubation period during which the fluorescently-labeled substrates may be modified enzymatically (step 2), unreacted substrates are removed using a washing step (step 3). Cells containing catalytically-active glycosyltransferases retain the fluorescent product inside the cell, now as part of a polysaccharide. Finally, desired cells (with high fluorescence, hence high-level polysaccharide production) are screened by rapid sorting techniques such as fluorescence activated cell sorting (FACS) (step 4), followed by sequencing (step 5)

In this approach, derivatives of natural substrates that are labeled with fluorescent groups are introduced into the culture medium of bacterial cells. They are substrates for desired polymerases involved in polysaccharide biosynthesis, and can enter and leave the cell freely through the cell membrane or pass through a transmembrane sugar transport protein. However, after transglycosylation occurs in the cytoplasm, the fluorescent groups are trapped in, and label, product polysaccharide molecules; these large fluorescently-labeled saccharides cannot pass through the cell membrane and are thus trapped in the cell. Meanwhile, any fluorescently-labeling substrate that did not participate in the polysaccharide synthesis can be removed using washing steps, so the intensity of the fluorescent signal in each cell is positively correlated with the cell’s ability to synthesize polysaccharide chains. Thus, the ability of individual cells within a cell library (domesticated or containing a specific gene mutation) to synthesize polysaccharide chains can be screened by high-throughput combined with rapid sorting techniques such as fluorescence activated cell sorting (FACS) (Fig. 1).

### Single-color labeling strategy

Based on this strategy, the directed evolution of a series of GTs related to bacterial polysaccharide synthesis was realized. In 2006, the strategy of fluorophore labeling was applied to the high-throughput screening of high-catalytic-efficiency mutants of bacterial sialyltransferase CstII, which was the first report of successful application of a screening method based on labeled substrate in the directed evolution of GTs [46]. CstII can catalyze the transfer of a negatively-charged *N*-acetylneuraminic acid (Neu5Ac) to a fluorescently-labeled neutral glycan to form a negatively-charged fluorescently-labeled product [47]. This product was retained inside bacterial cells. Based on this principle, single cells with strong intracellular fluorescence signals could be sorted by FACS, and these cells theoretically contained high-catalytic-efficiency CstII mutants. Finally, a mutant (in which the 91-position phenylalanine was replaced by tyrosine) with 400-fold higher catalytic efficiency than the wild-type was screened from a library of > 10^6^ CstII mutants.

### Dual-color labeling strategy

**Fig. 2:**
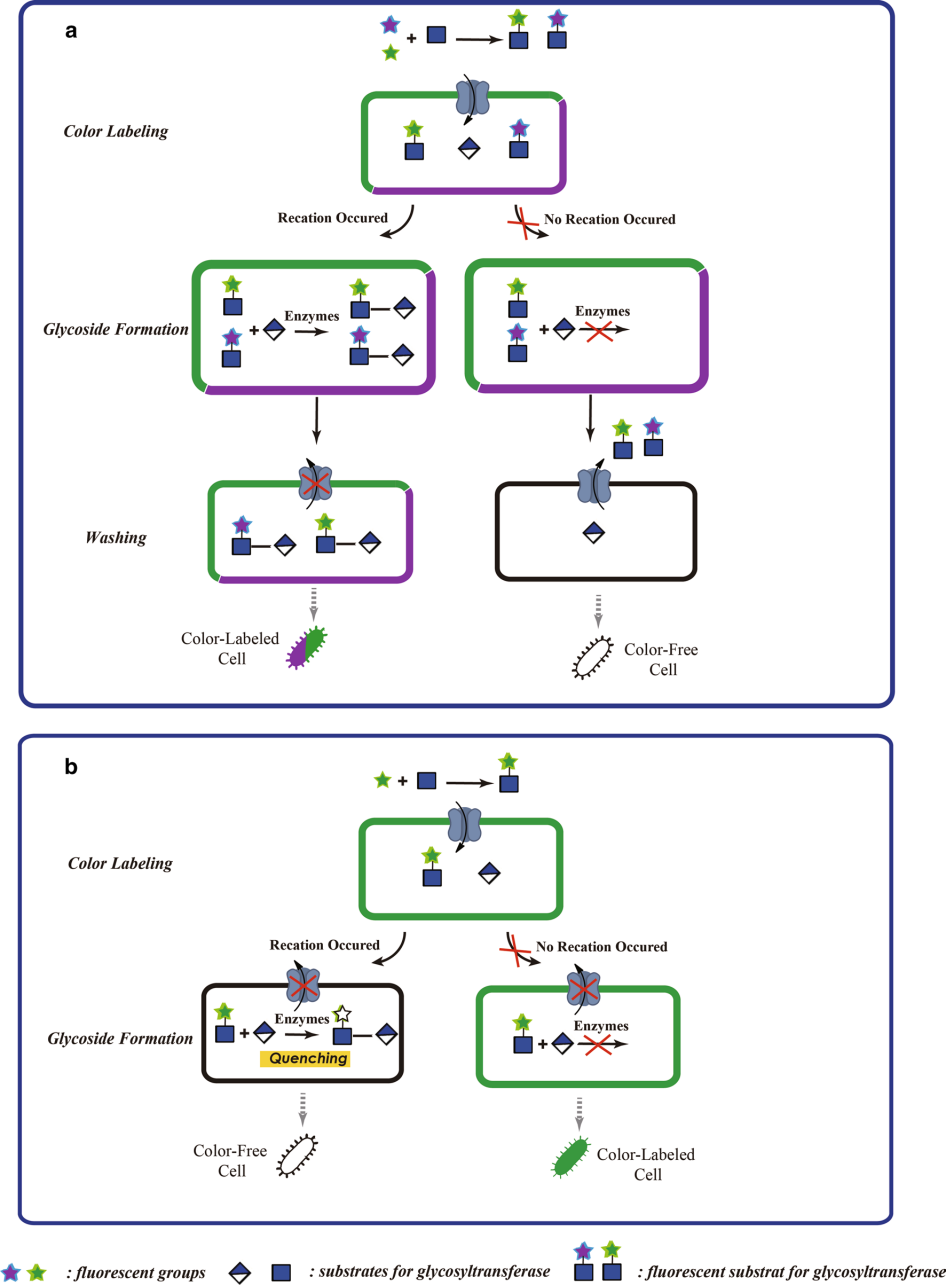
Schematic view of cell-based high-throughput screening strategies based on dual fluorescence labeling and fluorescence quenching. **a** In the dual fluorescence labeling method, two fluorescent substrates are employed simultaneously. The combined (“dual”) absorption of each fluorescent group is used for detection to minimize the probability of selecting for false-positive clones. **b** In the application of fluorescence quenching, the fluorescence signal of the carbohydrate chain is quenched once glycosyltransferases transfer the fluorescently-labeled substrate onto the acceptor, and then cells containing (highly) catalytically active enzymes can be sorted by their low fluorescence. Otherwise, the sorting process is consistent with that in Fig. 1

Subsequently, by upgrading the fluorescently-labeling substrate from monochromatic to dichromatic, the accuracy of the high-throughput screening method based on whole cells was significantly improved. Compared with single-color labeling, the dual-color labeling strategy in which some molecules of the substrate carry one “color” fluorophore and other molecules of the same substrate carry the other “color”, thereby minimizing the probability of selecting for false-positive clones by using the combined (“dual”) absorption for detection (Fig. 2a). This is clearly beneficial for the identification of highly active mutants. Yang et al. labeled *N*-acetylgalactosamine (GalNAc) analogues with a green fluorescent group (BODIPY) or a blue fluorescent group (coumarin); and these molecules could freely cross the cell membrane of bacteria, and were used together for the directed evolution of *β*-1, 3-galactosyltransferase CgtB [48]. Using uridine diphosphate galactose (UDP-Gal) as a donor, CgtB can transfer galactose to a GalNAc residue of a saccharide chain to form the Gal-*β*-1, 3-GalNAc structure. The transglycosyl products containing Gal residues cannot pass through the cell membrane, resulting in the accumulation of blue and green fluorescently-labeled products in the cell. Cells showing simultaneous enhancement of both fluorescence signals were screened by FACS, and a N26K/L68I/K151E/L227G/E234D mutant of CgtB, named CgtB-S42, with wider substrate specificity and higher catalytic efficiency than wild-type CgtB, was screened from the CgtB mutant library (> 10^7^ mutants). When UDP-glucose was used as the donor substrate, the catalytic efficiency of CgtB-S42 was at least 300-fold higher than that of the wild-type enzyme. In 2019, Tan et al*.* used the dichromatic-labeling FACS strategy to conduct high-throughput screening of a library of mutants of fucosyltransferase FutA (> 10^7^ mutants) [49]. This enzyme can transfer fucose to the C3–OH site of the GlcNAc group of *N*-acetyllactosamine (LacNAc). After three rounds of screening, an excellent mutant, S45F/D127N/R128E/H131I/Y199N/E340D/V368A, named FutA-M23, was identified. The catalytic efficiency of FutA-M23 in the synthesis of Lewis X and 3′-fucosyllactose was 6- and 14-fold higher than that of wild-type FutA, respectively.

### Fluorescence quenching strategy

In the method described above, the cell fluorescence signal is enhanced by the process of polysaccharide synthesis. In another effective method, the fluorescence signal of a saccharide chain may be quenched after transglycosylation reaction, and then mutant strains with enhanced polysaccharide synthesis ability can be isolated by reverse screening (Fig. 2b). For example, in 2007, Williams et al*.* used the fluorescence receptor quenching screening method to expand the receptor substrate specificity of the oleandomycin GT OleD through directed evolution [50]. Wild-type OleD only showed weak catalytic efficiency toward common phenolic receptors including the fluorophore 4-methylumbelliferone (MU), kaempferol and daidzein [51]. Once MU is glycosylated, bonding with one glucose residue, the C7–OH group of MU is masked and the fluorescence signal is weakened. Thus, the fluorescence signal of the whole cell was coupled with the catalytic efficiency of OleD mutants toward substrate MU, and high-throughput screening of an OleD mutant library was performed. Several mutants were screened and their mutation sites were analyzed; amino acids 67, 132, and 242 were found to be the key sites affecting the activity. Then, the amino acid mutations were combined to obtain an OleD mutant (P67T/S132F/A242V) whose *k*_cat_ toward MU was 30-fold higher than that of the wild-type enzyme. More surprisingly, the number of receptor substrates of OleD P67T/S132F/A242V observably expanded, which directly proves the feasibility of using similar strategies to expand the specificity of GT donors or receptor substrates. Besides increasing the catalytic efficiency, improving tolerance towards substrates is also valuable in GT research, and will directly expand the range of saccharide types that can be synthesized by enzymatic approaches.

### Screening toward a highly efficient polysaccharide biosynthesis based on fluorescently-labeled substrates—a perspective

The development of cell sorting technology makes it possible to screen individual clones with different fluorescence signals from a large number of bacterial cells. The screening efficacy of advanced flow cytometry can reach > 25,000 cells per second. Therefore, by establishing a relationship between the ability of a cell to synthesize bacterial polysaccharides and the number of fluorescent molecules accumulated in the cell, scientists can achieve high-throughput screening of individual cells in a mutant library that are able to efficiently synthesize carbohydrate chains. This can be achieved by artificial modification of a specific substrate, which has been successfully applied in the directed evolution of several GTs. It should be specifically pointed out that the screening method based on fluorescence-labeled substrate can not only be used to screen strains or enzyme molecules with better activity to synthesize polysaccharides, but can also be used to expand substrates pecificity of desired enzymes. The later strategy, usually named substrate engineering, is an effective way to enrich the diversity of carbohydrate chain structures that can be artificially prepared.

## Screening based on biosensors in vivo

**Fig. 3:**
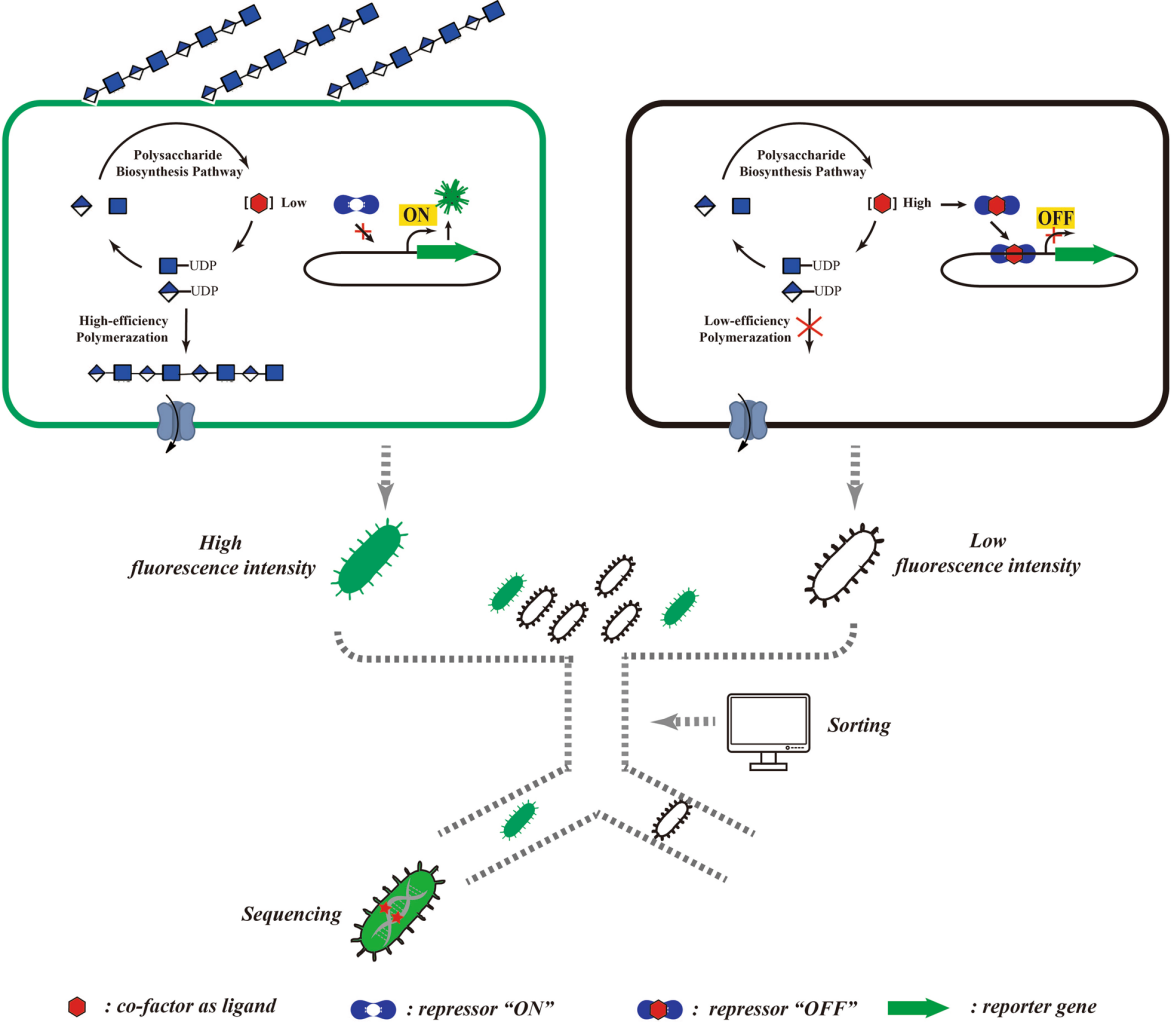
High-throughput screening of polysaccharide biosynthesis hosts based on biosensors in vivo. This strategy relies on linking the production of a fluorescent protein to the intracellular concentration of a target metabolite that is associated with the cell’s ability to synthesize polysaccharide. Then, high-yield strains can be enriched through FACS and sequenced

Biosensors are one of best too s of synthetic biology, both for high-throughput screening and for direct optimization of biosynthesis pathways. The synthesis of bacterial polysaccharides involves a number of continuous enzymatic reactions, and the level of synthesis can be characterized by the concentration of specific substances in cells, such as intermediates or energy substances. In theory, biosensors of these intermediate substrates or products can be used for high-throughput screening and identification of polysaccharide synthesis levels within a single cell, to enable the optimization of synthesis pathways and directional evolution of synthase proteins [52–56] (Fig. 3). Biosensors comprise a sensor part (e.g. riboswitches, ribozymes, transcription factors, enzymes, or periplasmic-binding proteins) and an actuator part (e.g. fluorescent reporters, regulatory switches, or selection markers) [57]. Upon binding of the effector molecule, conformational change of the sensor part controls the expression of the actuator part, which affects the phenotype of the bacteria. This phenotype can be expressed in different fluorescence values, different tolerance to antibiotics, or different growth states in a nutritionally-deficient environment.

### Biosensors for intermediates involved in the polysaccharide biosynthesis

For polysaccharide biosynthesis, special emphasis should be placed on biosensors of substances related to carbohydrate chain synthesis and energy metabolism. Nucleotide activation and energization of monosaccharide donors is a prerequisite for GTs to recognize and complete monosaccharide transfer, making bacterial polysaccharide synthesis a significantly energy-consuming process. Taking hyaluronic acid as an example, for each molecule of disaccharide repeat unit synthesized, the cell consumes five molecules of ATP, two molecules of NADH, and one molecule of acetyl-CoA [58]. In 2014, San Martín et al*.* constructed a Förster Resonance Energy Transfer (FRET) sensor to detect the concentration of pyruvate in cells in real time [59]. The FRET biosensor has the advantages of good orthogonality, high time-resolution, and it was easy to construct. Pyruvate is involved in the mutual transformation between monosaccharides, fats, and amino acids through the acetyl-CoA and tricarboxylic acid cycles [60]. However, although the FRET sensor is very suitable for monitoring the metabolic kinetics of intracellular pyruvate, it has not yet been reported that the system can be used to analyze bacterial polysaccharide synthesis.

### Biosensors for coenzymes involved in the polysaccharide biosynthesis

A biosensor for detecting NADH concentration can be designed by coupling a promoter responsive to change of the reduced/oxidized coenzyme concentration ratio to a reporter gene system. Siedler et al*.* developed an NADPH/NADP^+^ redox biosensor in *Escherichia coli* using natural redox-sensitive transcription factor (TF) SoxR in 2013 and applied it to sort mutants of NADPH-dependent alcohol dehydrogenase from *Lactobacillus brevis* (LbAdh) that showed improved activity toward the substrate 4-methyl-2-pentanone [61]. In 2014, Knudsen et al*.* designed a TF sensor in *Saccharomyces cerevisiae* that can respond to the NADH/NAD^+^ ratio [62]. Glycerol-3-phosphate dehydrogenase, encoded by the gene *GPD2*, plays a central role in redox metabolism in *S. cerevisiae*. In anaerobic conditions, due to the reduction of dihydroxyacetone phosphate to glycerol-3-phosphate by NADH-coupled dihydroxyacetone phosphate, the expression of *GPD2* increases with the increase of NADH oxidation demand. Therefore, the transcriptional level of a GFP reporter gene under the control of the promoter of *GPD2* was related to the concentration of NADH.

### Biosensors for monosaccharides

A biosensor responsive to monosaccharide molecules has been designed and used in the domestication of monosaccharide-producing strains. In 2013, Cho obtained a Neu5Ac aptazyme with high specificity and affinity by screening in vitro. A biosensor based on the aptazyme can monitor the concentration of Neu5Ac in real time [63]. In 2017, Yang designed a directed evolution strategy for Neu5Ac synthesis based on the above-mentioned Neu5Ac aptazyme [64]. Through fusion of the Neu5Ac aptazyme and gene *tetA* (the expression of *tetA* increases the sensitivity of bacteria to Ni^2+^), the concentration of Neu5Ac was coupled with the growth rate of a strain. The authors conducted directed evolution of a ribosome-binding site library and finally increased the yield of Neu5Ac by 34%.

### Biosensors for polysaccharide biosynthetic ability monitoring—a perspective

**Fig. 4:**
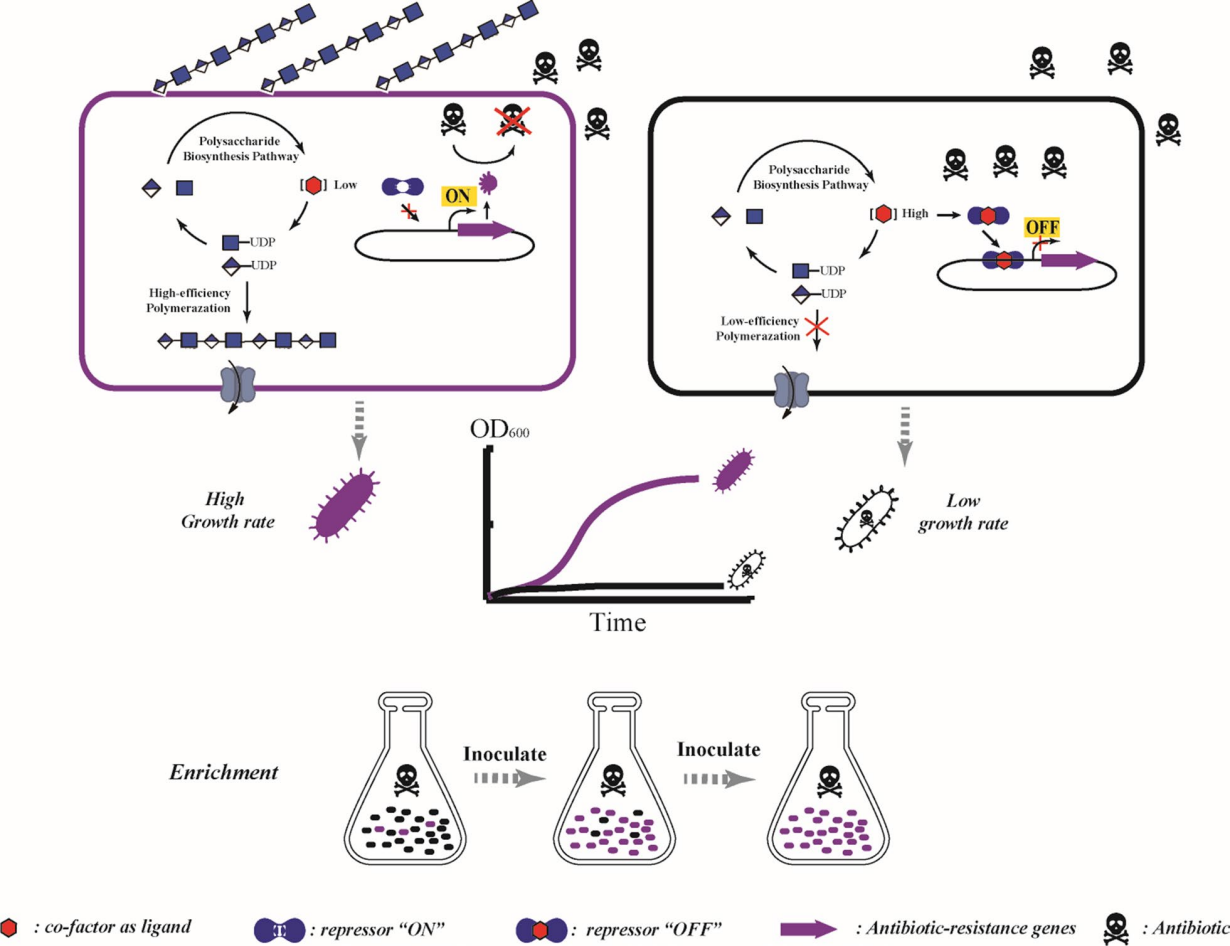
Cell-based high-throughput screening based on the coupling of cell growth with in vivo polysaccharide biosynthesis. An in vivo active biosensor responds to the intracellular concentration of a target metabolite that is associated with the cell’s ability to synthesize polysaccharides. One selects a suitable actuator (e.g., antibiotic, auxotroph, or toxin) that can directly select for and enrich high-yield strains. In selection conditions, the polysaccharide biosynthesis level is coupled with cell growth, so one can isolate cells with high productivity that also synthesize polysaccharides at a high level

Biosensors are one of the fastest developing synthetic biology tools, and can be used for single cell recognition to achieve ultra-high-throughput screening. Although there have been many reports on the application of biosensor systems in response to molecules related to polysaccharide synthetic pathways, and they have been successfully applied to the optimization of monosaccharide synthesis pathways, they have not yet been used directly in the optimization of polysaccharide synthesis. However, biosensors have great application potential in the preparation of bacterial polysaccharides, and their application will expand to the construction and optimization of high-yield strains and the exploration and directed evolution of high-catalytic-efficiency enzymes. In addition, with the development of quorum sensing technology, screening efficiency based on biosensor systems will be significantly improved by coupling detection of ability to biosynthesize polysaccharides with cell growth rate, thus enabling isolation of cells with high productivity that also contain enzyme mutants with high catalytic efficiency (Fig. 4).

## Conclusions

Bacterial polysaccharides have extensive applications in many fields [7–16]. It is difficult to obtain a carbohydrate chain of > 12 monosaccharide residues by chemical, in vitro enzymatic, or chemoenzymatic methods. Only the use of cell factories (microbial fermentation) is suitable for the preparation of long polysaccharides. However, polysaccharide biosynthesis strains, especially engineered strains in which polysaccharide biosynthesis pathways are artificially introduced, must balance cell growth and product synthesis. It is necessary to regulate and reform the metabolic pathways to remove the rate-limiting factors in the process of polysaccharide synthesis to increase the yield. Using whole cell factories, large scale genetic mutation, and ultra-high-throughput screening of polysaccharide synthesis ability, the rate-limiting factors of polysaccharide synthesis can be found from a large number of clones with different phenotypes, and optimization of energy distribution can be used to solve the key problems. Therefore, the development of effective high-throughput approaches, based on biosensors for polysaccharide biosynthetic ability monitoring or fluorescently-labeled substrates, for cell sorting of a library that can be applied during the domestication of industrial engineered strains and metabolic pathway optimization of polysaccharide synthesis in engineered cells have important application value. Additionally, cell-based screening approaches are also suitable for the directed evolution of a single key enzyme in a polysaccharide biosynthesis pathway. Moreover, the screening of cells is a direct overall analysis of the cell factory and can realize the discovery and optimization of the limiting factors of intracellular polysaccharide synthesis, which is not possible in single-factor research systems in vitro. Here, this review provides an introduction to the methodologies of using cell-based high-throughput screening for desired polysaccharide-biosynthesizing cells, followed by a brief discussion of potential applications of these approaches in glycoengineering.

## Data Availability

Not applicable.

## Abbreviations

GAG: Glycosaminoglycan
HA: Hyaluronic acid
GT: Glycosyltransferases
HT: High-throughput
FACS: Fluorescence activated cell sorting
MU: 4-Methylumbelliferone
Neu5Ac: *N*-Acetylneuraminic acid
GalNAc: *N*-Acetylgalactosamine
UDP: Uridine diphosphate
Gal: Galactose
LacNAc: *N*-Acetyllactosamine
FRET: Förster Resonance Energy Transfer
TF: Transcription factor
G3P: Glycerol-3-phosphate
DHAP: NADH-coupled dihydroxyacetone phosphate
